# Supplementary material for: Urbanization and agricultural intensification destabilize animal communities differently than diversity loss
Source: Nat Commun. 2020 Jun 1;11:2686. doi: 10.1038/s41467-020-16240-6 (PMC7264125; doi:10.1038/s41467-020-16240-6)
Supplement: Supplementary file 1 — Supplementary Information [file 41467_2020_16240_MOESM1_ESM.pdf]

Urbanization and agricultural intensification destabilize animal communities differently than diversity loss

Olivier et al.

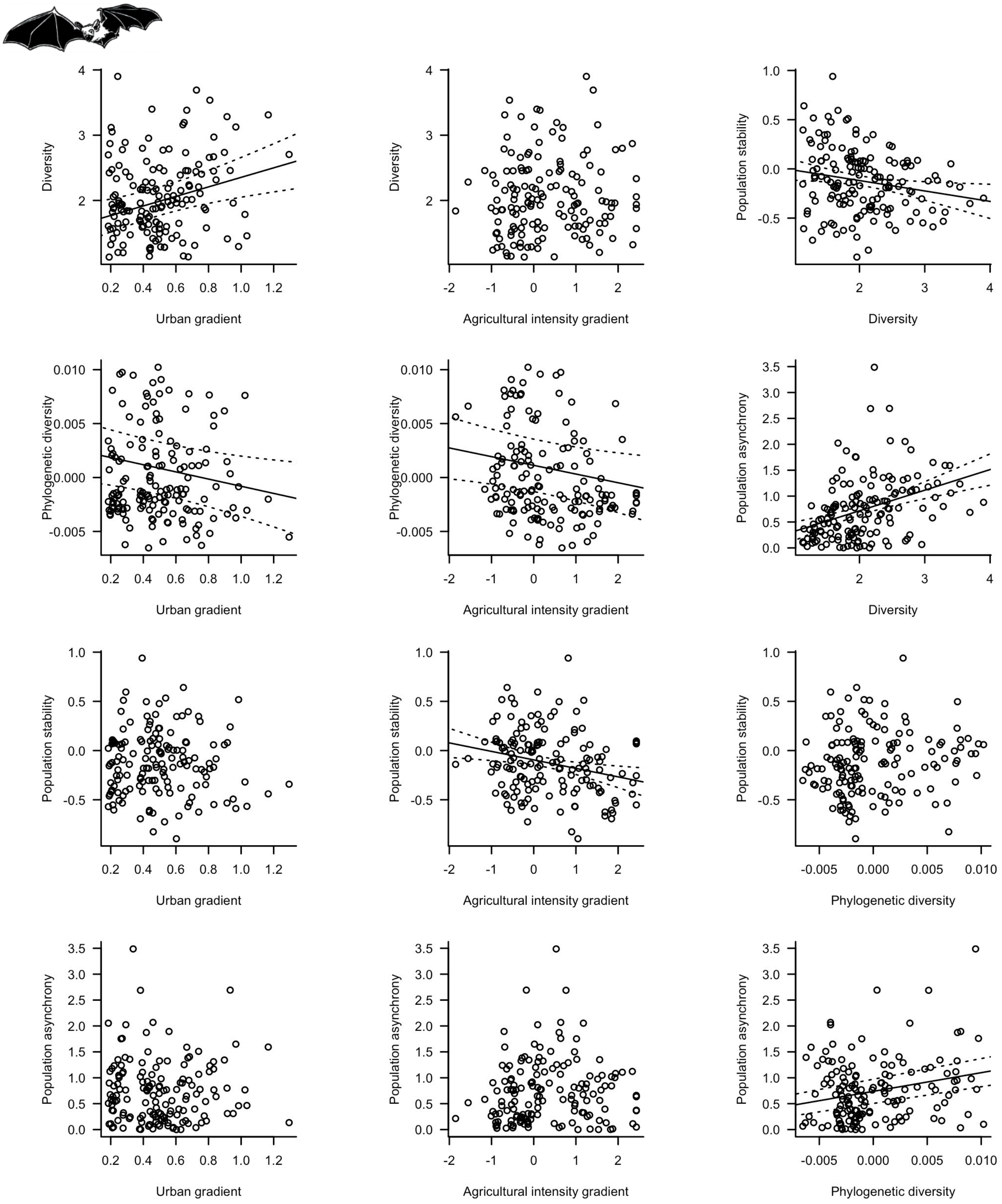

Supplementary Fig. 1. Relationships tested in the structural equation model used for bat communities. Significant relationships are represented by solid and dotted lines (see estimates and *p*-values in Supplementary Table 3). Solid lines represent linear mixed-effects model predictions for significant relationships. Dotted lines represent the CI 95% of model predictions. Population stability (measured as weighted mean population stability), asynchrony and urban gradient are log-transformed.

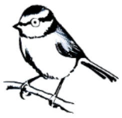

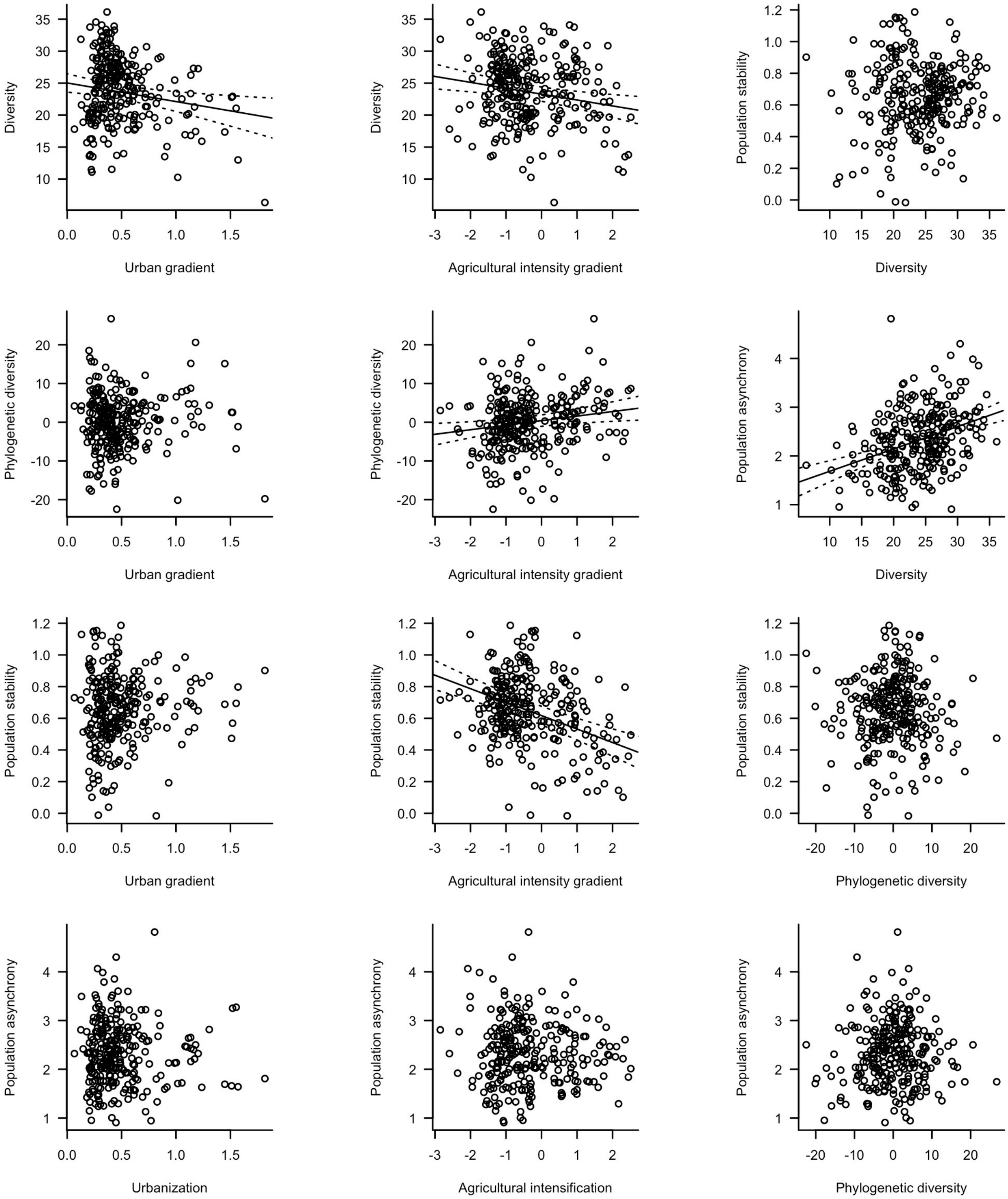

Supplementary Fig. 2. Relationships tested in the structural equation model used for bird communities. Significant relationships are represented by solid and dotted lines (see estimates and *p*-values in Supplementary Table 3). Solid lines represent linear model predictions for significant relationships. Dotted lines represent the CI 95% of model predictions. Population stability (measured as weighted mean population stability), asynchrony and urban gradient are log-transformed.

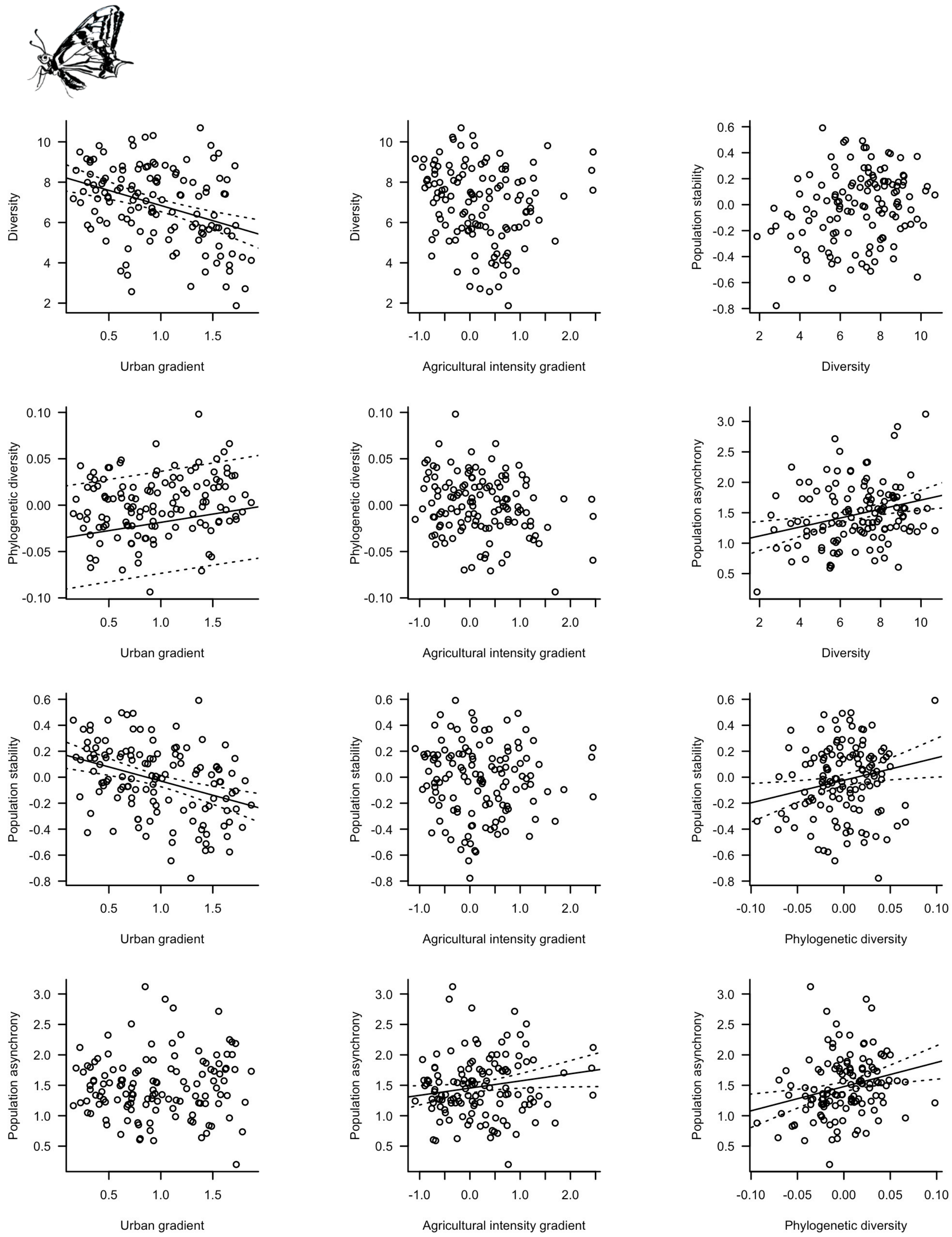

Supplementary Fig. 3. Relationships tested in the structural equation model used for butterfly communities. Significant relationships are represented by solid and dotted lines (see estimates and *p*-values in Supplementary Table 3). Solid lines represent linear model predictions for significant relationships. Dotted lines represent the CI 95% of model predictions. Population stability (measured as weighted mean population stability), asynchrony and urban gradient are log-transformed.

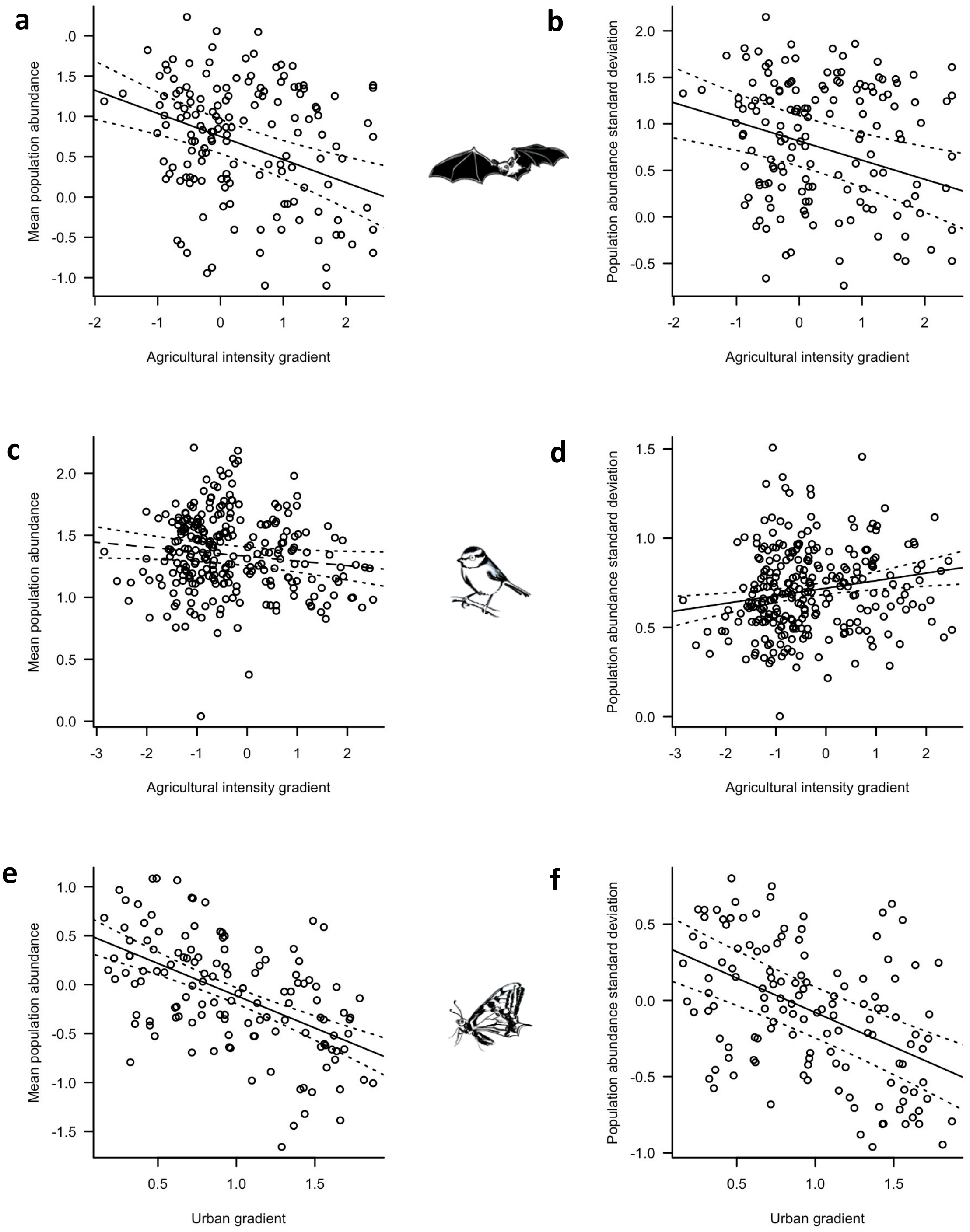

Supplementary Fig. 4. Relationships between habitat degradation and the community mean population abundance and the average standard deviation of population abundances for bat (a-b, estimate = -0.29, *p*-value = 10^{-4} and estimate = -0.20, *p*-value = 0.001 respectively), bird (c-d, estimate = -0.04, *p*-value = 0.051 and estimate = 0.04, *p*-value = 0.003 respectively) and butterfly (e-f, estimate = -0.66, *p*-value = 3.17×10^{-11} and estimate = -0.45, *p*-value = 3.5×10^{-9} respectively) communities. Solid lines represent model predictions for significant relationships, except for (c) where predictions are a dashed line because of a marginal significancy. Dotted lines represent the CI 95% of model predictions. Linear mixed-effects models are used for bats, and linear models for birds and butterflies. The community mean population abundance and the average standard deviation of population abundances are log-transformed.

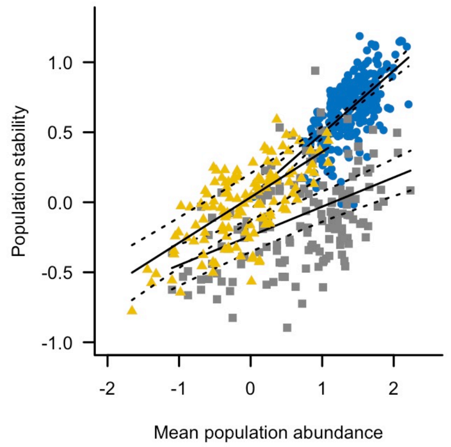

Supplementary Fig. 5. Relationships between population stability measured as weighted mean CV of population abundances and community mean population abundance for bird, bat and butterfly communities. Bats are grey squares, birds blue circles and butterflies yellow triangles. Black lines represent model predictions and dotted lines represent the CI 95% of model predictions (estimate = 0.45, *p*-value = 4.8×10^{-10}, estimate = 0.21, *p*-value = 10^{-30}, estimate = 0.32, *p*-value = 8×10^{-20} for bat, bird and butterfly communities respectively). Linear mixed-effects models are used for bats, and linear models for birds and butterflies. Population stability and community mean population abundance are log-transformed.

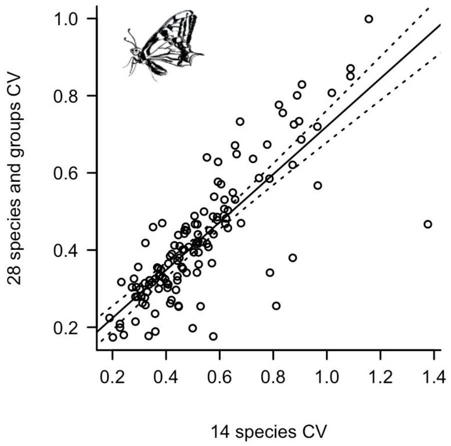

Supplementary Fig. 6. Relationship between the coefficients of variation of the total abundance of the butterfly communities including all species and species groups and the butterfly communities restricted to the 14 species that could be identified with certainty (estimate = 0.62, *p*-value = 2×10^{-16}). Black line represents linear model predictions and dotted lines represent the CI 95% of model predictions.

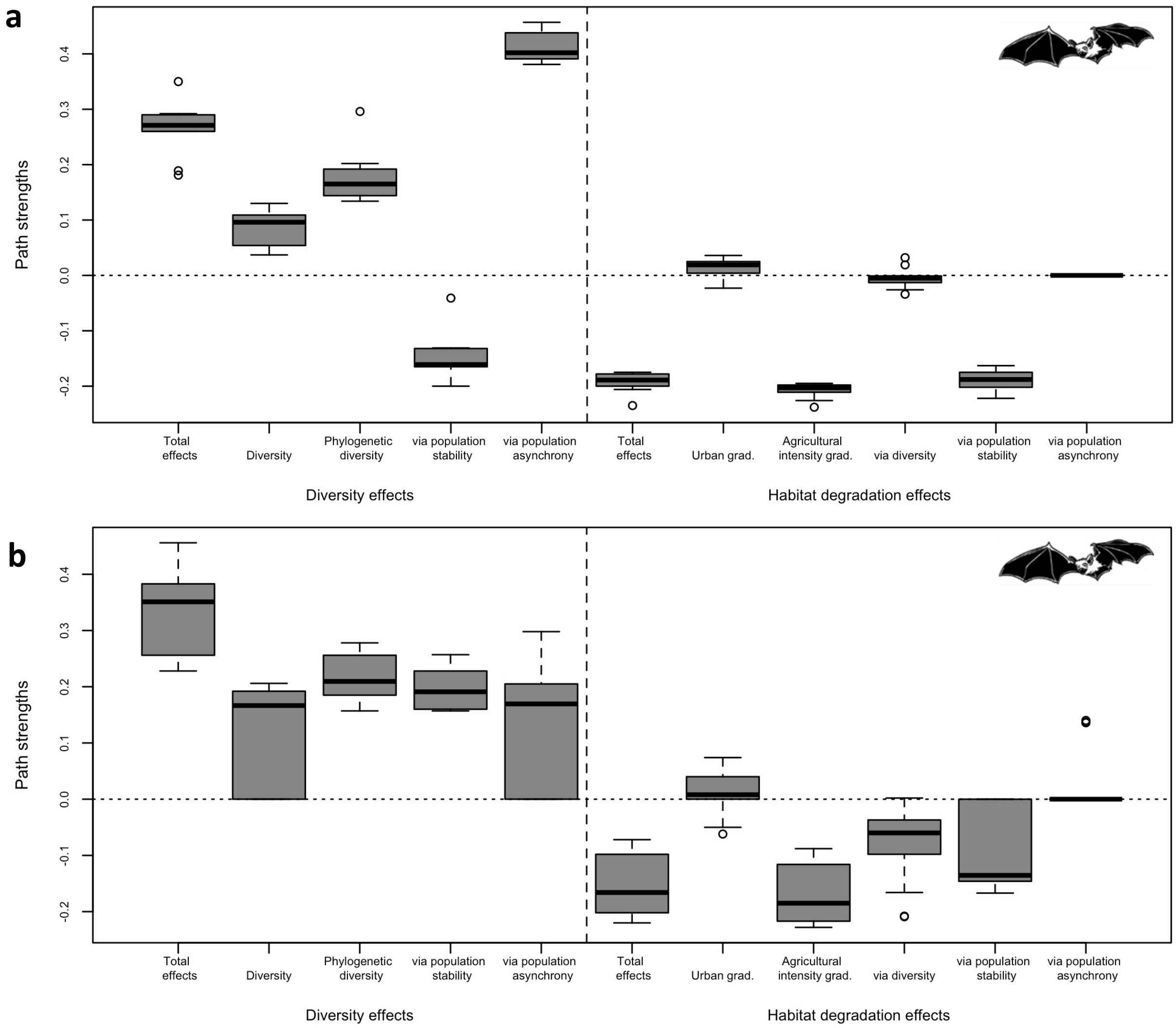

Supplementary Fig. 7. Boxplots of the strength of the different paths by which diversity and habitat degradation affect the stability of local communities of bats for the three buffer sizes (250, 500 and 1000m) and the three data selections (Datasets 1, 2 and 3). **a.** Distributions of path strengths from n=9 SEM models using the Shannon diversity index and residual weighted MPD as diversity and phylogenetic diversity measures. **b.** Distributions of path strengths from n=18 SEM models using species richness and residual non-weighted MPD (9 models) as well as species richness estimated by the Chao index and residual non-weighted MPD (9 models). As in Table 1, the strength of each path is calculated from the standardized coefficients of the structural equation models, multiplying coefficients along a path and summing the results over the different paths. Total effects refer to the sum of the effects by which diversity and phylogenetic diversity, or urbanization and agricultural intensification, affect community stability. Effects via a variable are the sum of the effects of diversity or habitat degradation on community stability that are channelled by a direct effect on this variable. Null values stand for non-significant effects. For each boxplot: center line, median; box limits, upper and lower quartiles; whiskers, 1.5x interquartile range; points, outliers.

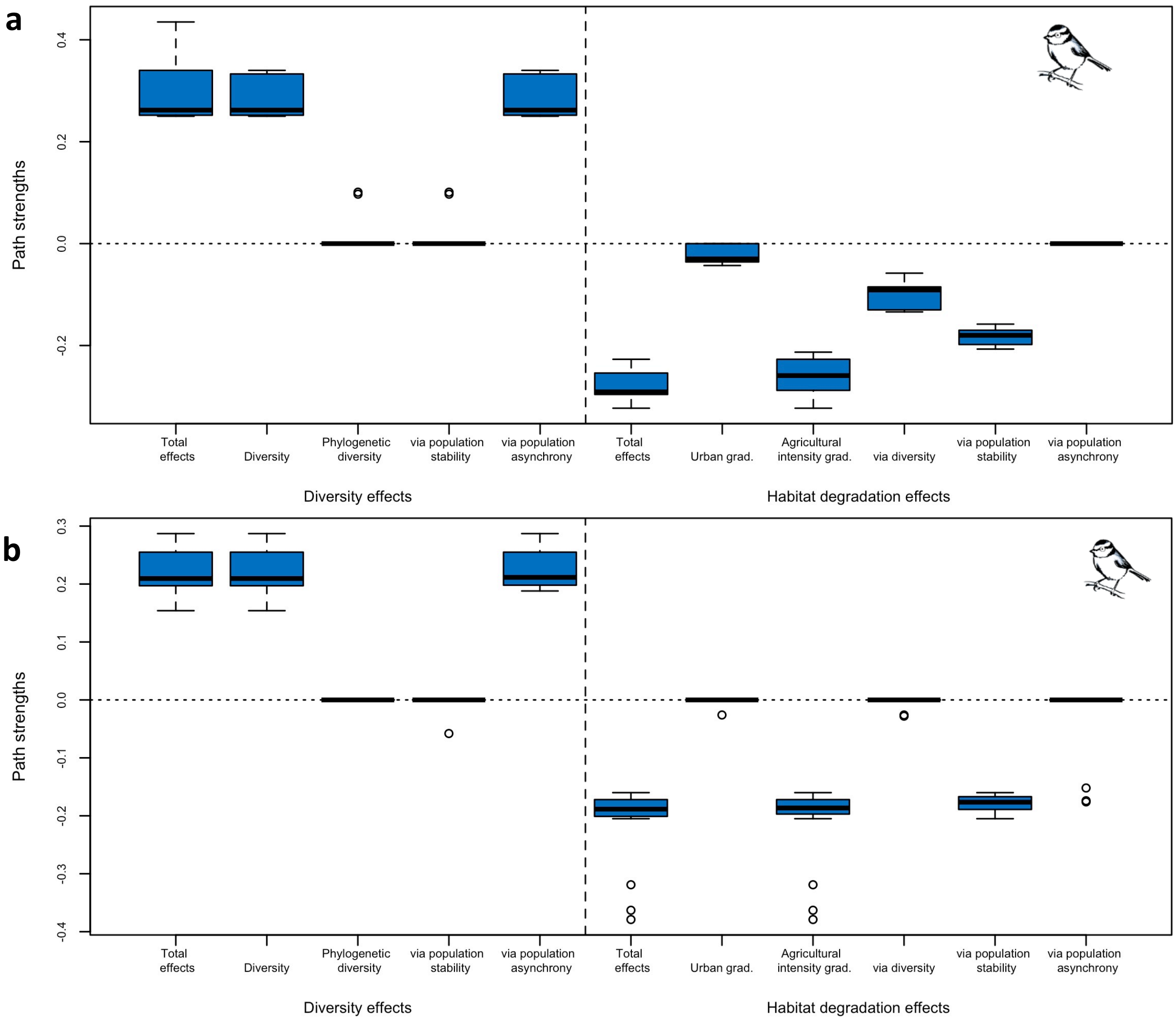

Supplementary Fig. 8. Boxplots of the strength of the different paths by which diversity and habitat degradation affect the stability of local communities of birds for the three buffer sizes (250, 500 and 1000m) and the three data selections (Datasets 1, 2 and 3). **a.** Distributions of path strengths from n=9 SEM models using the Shannon diversity index and residual weighted MPD as diversity and phylogenetic diversity measures. **b.** Distributions of path strengths from n=18 SEM models using species richness and residual non-weighted MPD (9 models) as well as species richness estimated by the Chao index and residual non-weighted MPD (9 models). As in Table 1, the strength of each path is calculated from the standardized coefficients of the structural equation models, multiplying coefficients along a path and summing the results over the different paths. Total effects refer to the sum of the effects by which diversity and phylogenetic diversity, or urbanization and agricultural intensification, affect community stability. Effects via a variable are the sum of the effects of diversity or habitat degradation on community stability that are channelled by a direct effect on this variable. Null values stand for non-significant effects. For each boxplot: center line, median; box limits, upper and lower quartiles; whiskers, 1.5x interquartile range; points, outliers.

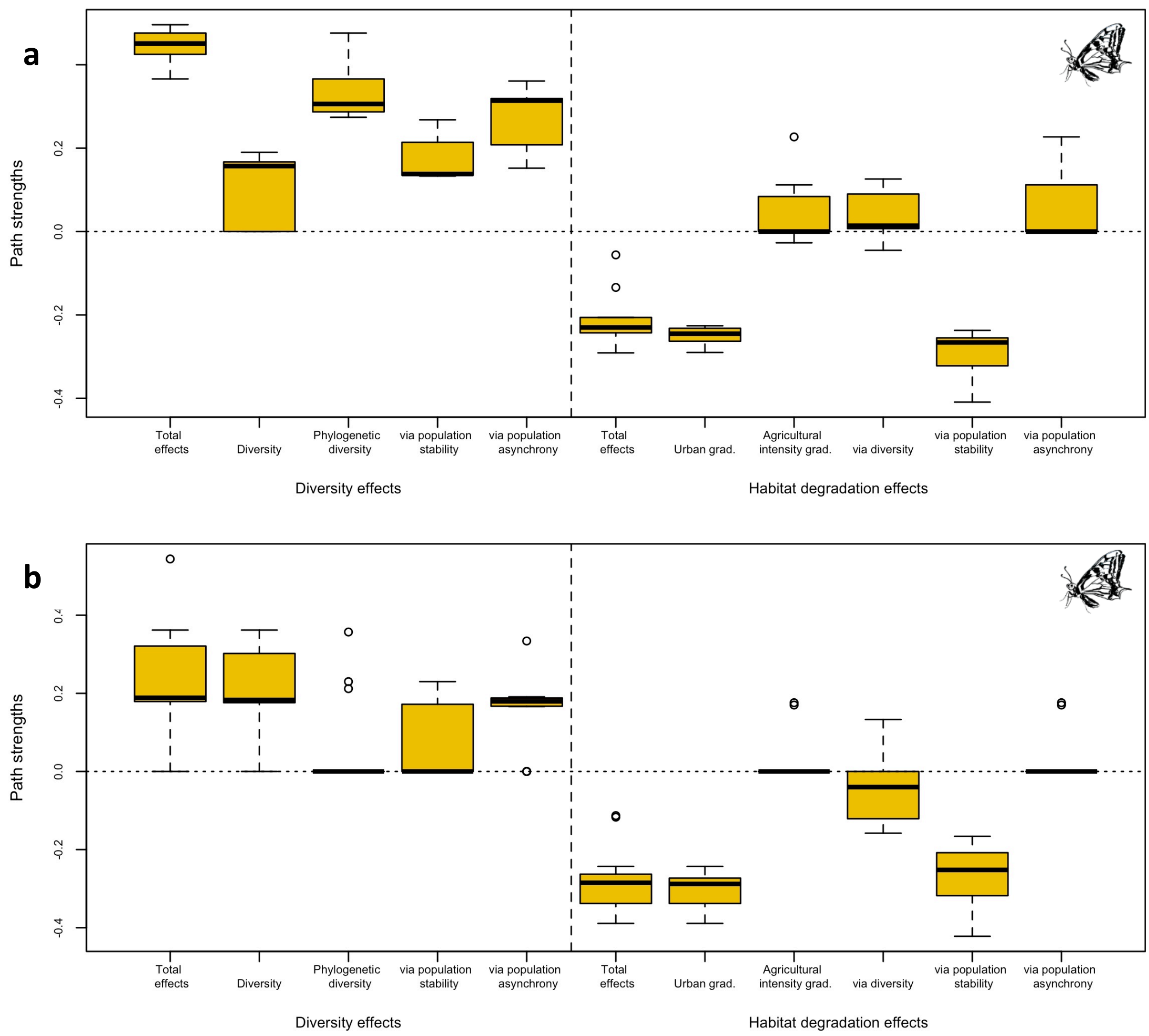

Supplementary Fig. 9. Boxplots of the strength of the different paths by which diversity and habitat degradation affect the stability of local communities of butterflies for the three buffer sizes (250, 500 and 1000m) and the three data selections (Datasets 1, 2 and 3). **a.** Distributions of path strengths from n=9 SEM models using the Shannon diversity index and residual weighted MPD as diversity and phylogenetic diversity measures. **b.** Distributions of path strengths from n=18 SEM models using species richness and residual non-weighted MPD (9 models) as well as species richness estimated by the Chao index and residual non-weighted MPD (9 models). As in Table 1, the strength of each path is calculated from the standardized coefficients of the structural equation models, multiplying coefficients along a path and summing the results over the different paths. Total effects refer to the sum of the effects by which diversity and phylogenetic diversity, or urbanization and agricultural intensification, affect community stability. Effects via a variable are the sum of the effects of diversity or habitat degradation on community stability that are channelled by a direct effect on this variable. Null values stand for non-significant effects. For each boxplot: center line, median; box limits, upper and lower quartiles; whiskers, 1.5x interquartile range; points, outliers.

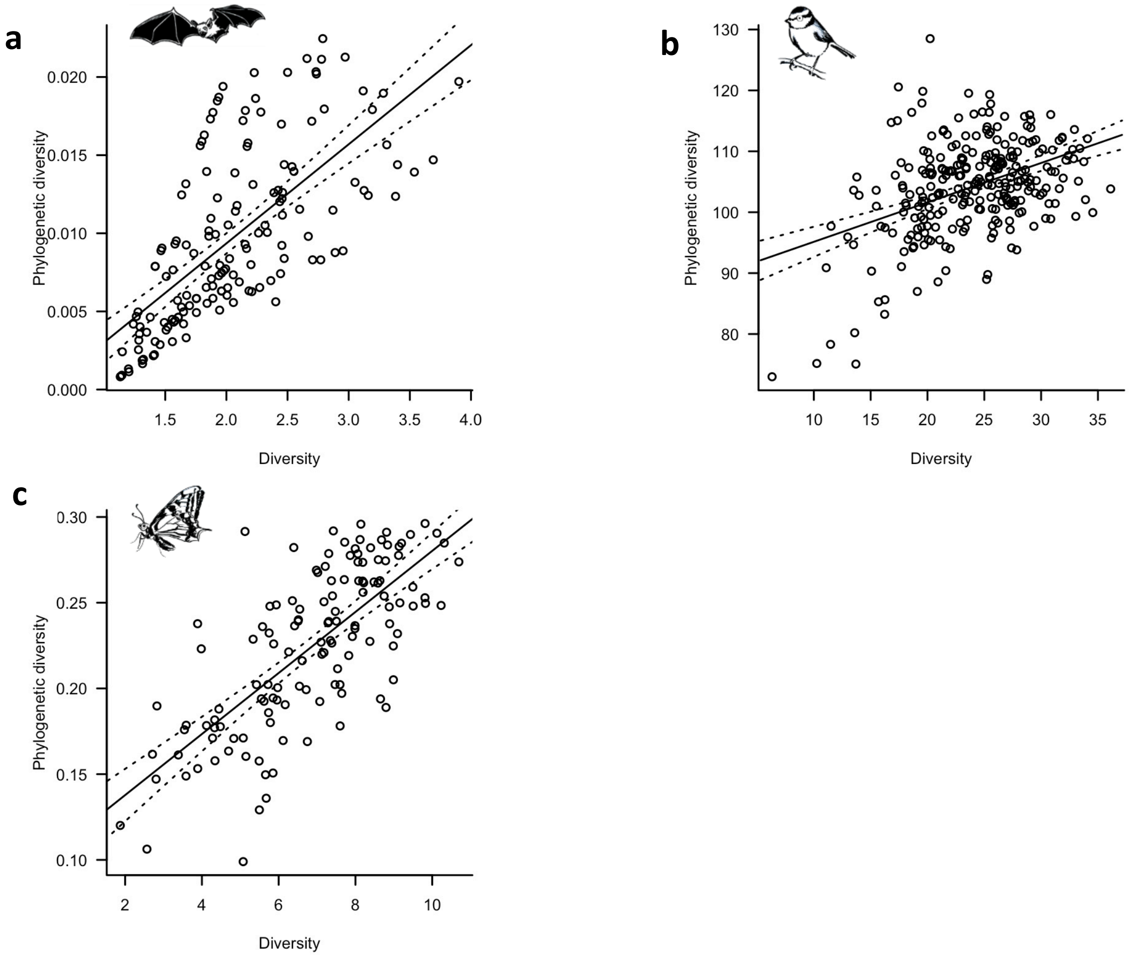

Supplementary Fig. 10. Relationship between species diversity and weighted phylogenetic diversity (Weighted Mean Pairwise Distance) for local communities of (a) bats (estimate = 0.01, *p*-value = 2×10^{-16}), (b) birds (estimate = 0.64, *p*-value = 5.14×10^{-13}) (c) and butterflies (estimate = 0.02, *p*-value = 2×10^{-16}). Black lines represent linear model predictions for significant relationships and dotted lines represent the CI 95% of model predictions. The Weighted Mean Pairwise Distance is in million years for birds and in number of substitutions per nucleotide for bats and butterflies.

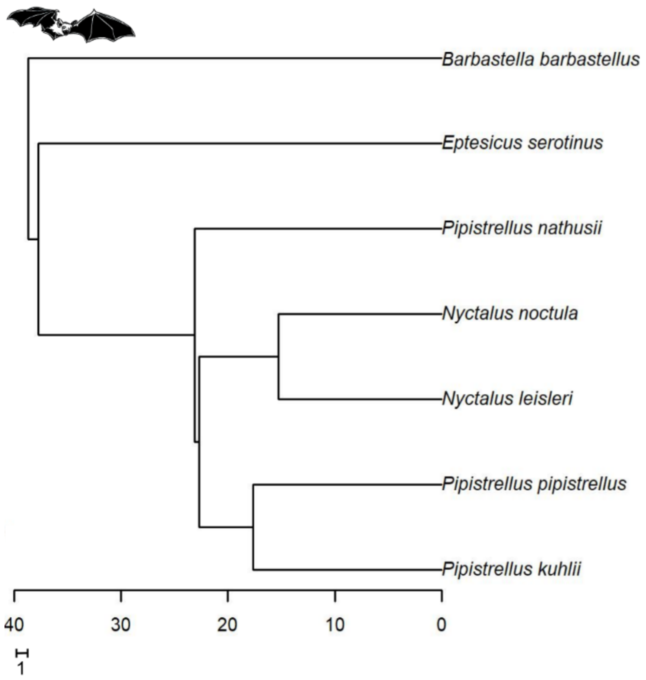

Supplementary Fig. 11. Phylogenetic tree of bat species present in our communities, based on Shi & Rabowski[1]. Branch lengths are in number of substitutions per nucleotide.

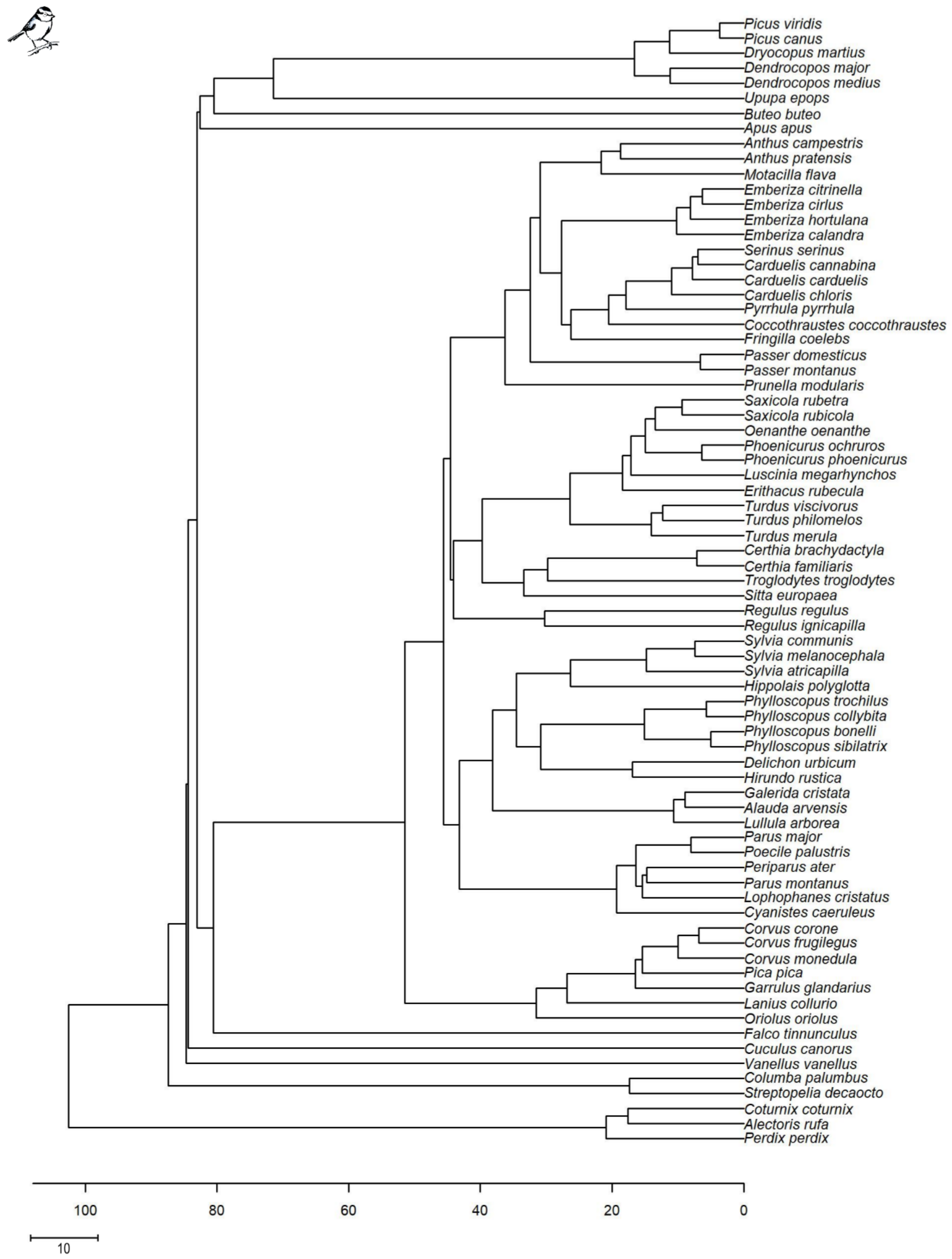

Supplementary Fig. 12. Phylogenetic tree of bird species present in our communities. Branch lengths are in millions years.

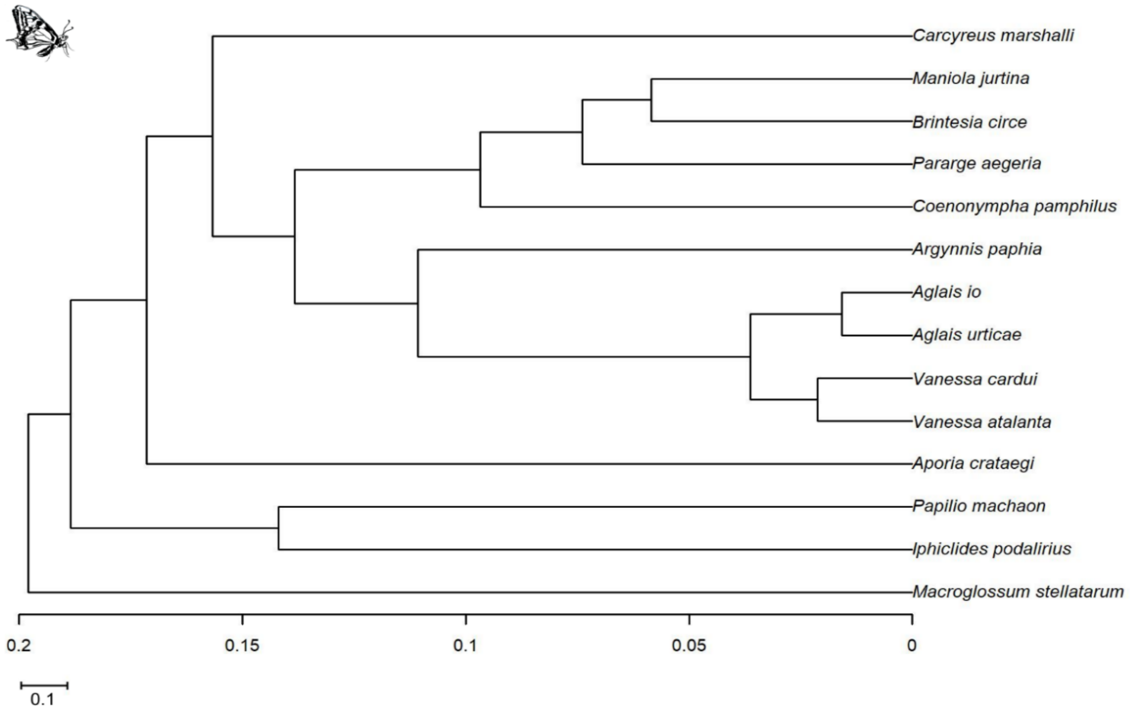

Supplementary Fig. 13. Phylogenetic tree of butterfly species present in our communities. Branch lengths are in number of substitutions per nucleotide.

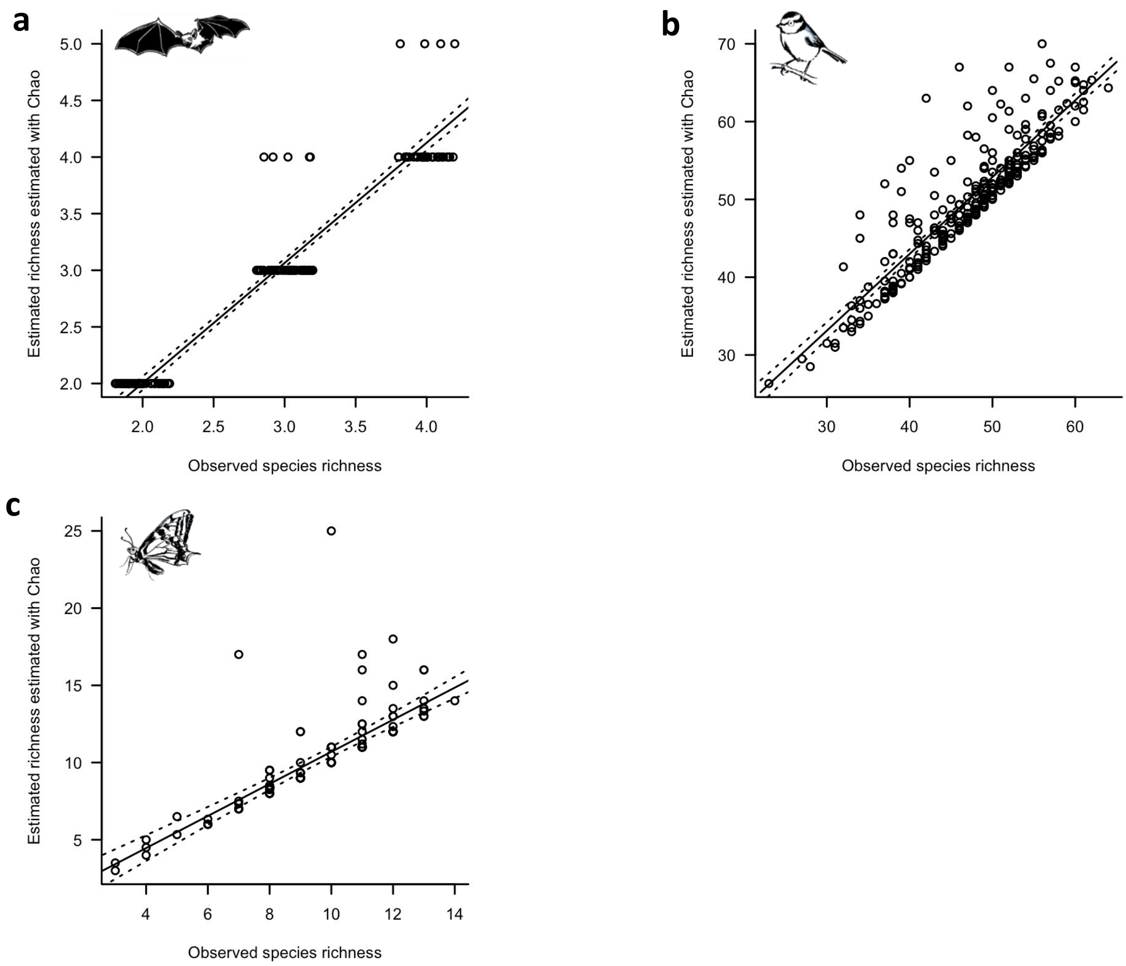

Supplementary Fig. 14. Relationships between species richness and Chao index for local communities of (a) bats (estimate = 1.06, p-value = 2×10^{-16}), (b) birds (estimate = 0.98, p-value = 2×10^{-16}) (c) and butterflies (estimate = 1.04, p-value = 2×10^{-16}). Black lines represent linear model predictions for significant relationships and dotted lines represent the CI 95% of model predictions.

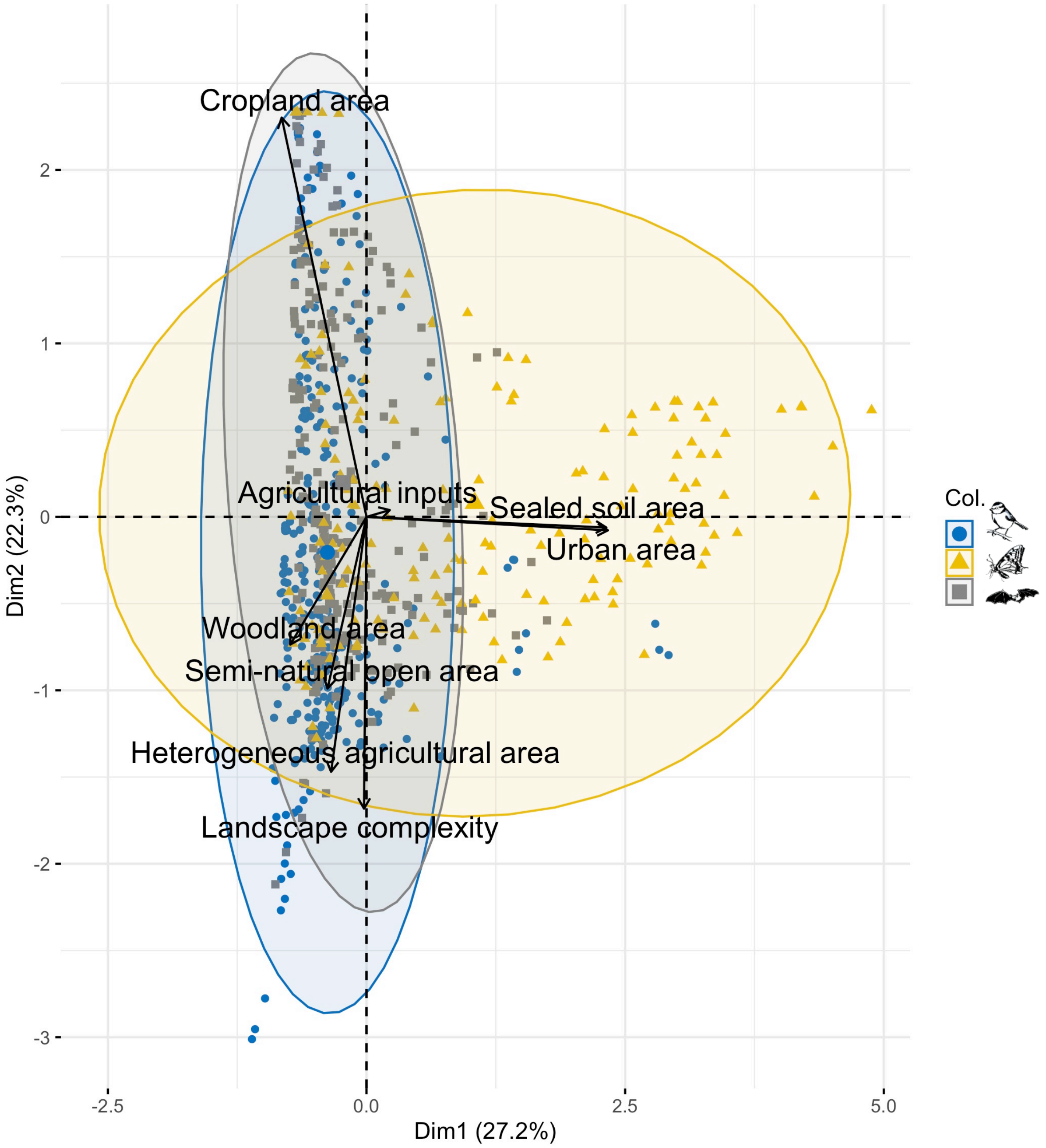

Supplementary Fig. 15. Graphical representation of the 2 first axis of the Principal Component Analysis for dataset with the longest time series without gap. Each point is a site and each ellipse contains 95% of the sites of the corresponding colored taxa. Each axis is a linear combination of the explanatory variables. Each arrow corresponds to the contribution of the variable to the two axes.

Supplementary Table 1. Species for bat, bird and butterfly local communities.

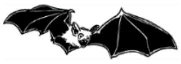	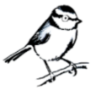		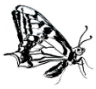
Nyctalus leisleri	*Coturnix coturnix*	*Regulus ignicapilla*	*Macroglossum stellatarum*
Nyctalus noctula	*Alectoris rufa*	*Regulus regulus*	*Iphiclides podalirius*
Eptesicus serotinus	*Perdix perdix*	*Phylloscopus bonelli*	*Papilio machaon*
Pipistrellus pipistrellus	*Buteo buteo*	*Phylloscopus collybita*	*Aporia crataegi*
Pipistrellus nathusii	*Vanellus vanellus*	*Phylloscopus sibilatrix*	*Cacyreus marshalli*
Pipistrellus kuhlii	*Columba palumbus*	*Phylloscopus trochilus*	*Argynnis paphia*
Barbastella barbastellus	*Streptopelia decaocto*	*Hippolais polyglotta*	*Aglais io*
	Cuculus canorus	*Sylvia atricapilla*	*Aglais urticae*
	Apus apus	*Sylvia communis*	*Vanessa atalanta*
	Upupa epops	*Sylvia melanocephala*	*Vanessa cardui*
	Dendrocopos major	*Erithacus rubecula*	*Pararge aegeria*
	Dendrocopos medius	*Luscinia megarhynchos*	*Coenonympha pamphilus*
	Dryocopus martius	*Phoenicurus ochruros*	*Maniola jurtina*
	Picus canus	*Phoenicurus phoenicurus*	*Brintesia circe*
	Picus viridis	*Saxicola rubetra*	
	Falco tinnunculus	*Saxicola rubicola*	
	Lanius collurio	*Oenanthe oenanthe*	
	Oriolus oriolus	*Turdus merula*	
	Garrulus glandarius	*Turdus philomelos*	
	Pica pica	*Turdus viscivorus*	
	Corvus corone	*Prunella modularis*	
	Corvus frugilegus	*Motacilla flava*	
	Corvus monedula	*Anthus campestris*	
	Lullula arborea	*Anthus pratensis*	
	Alauda arvensis	*Emberiza calandra*	
	Galerida cristata	*Emberiza cirlus*	
	Hirundo rustica	*Emberiza citrinella*	
	Delichon urbicum	*Emberiza hortulana*	
	Periparus ater	*Fringilla coelebs*	
	Lophophanes cristatus	*Coccothraustes coccothraustes*	
	Poecile palustris	*Pyrrhula pyrrhula*	
	Cyanistes caeruleus	*Carduelis cannabina*	
	Parus major	*Carduelis carduelis*	
	Parus montanus	*Carduelis chloris*	
	Sitta europaea	*Serinus serinus*	
	Certhia familiaris	*Passer domesticus*	
	Certhia brachydactyla	*Passer montanus*	
	Troglodytes troglodytes		

Supplementary Table 2. Definition and corresponding equations of the variables used to describe the diversity and the stability of ecological communities used in the path analysis.

Variables	Definition	Equation
Temporal stability of community total abundance	Reflects the amplitude of inter-annual variations in the total abundance of the community in a site.	$\frac{1}{\mathrm{CV}} = \frac{\mu}{\sigma}$
Weighted mean population stability	Mean amplitude of inter-annual variations of population abundances in a community, weighted by their relative abundance.	$\frac{1}{\overline{\overline{\mathrm{CV}_w}}} = \sum_i \frac{\mu}{\mu_i}\frac{\mu_i}{\sigma_i}$
Population asynchrony	Reflects the negative correlation degree among species temporal abundance fluctuations in a community.	$\frac{1}{\varphi} = \frac{(\sum_i \sigma_i)^2}{\sigma^2}$
Shannon diversity	Species diversity measure that takes species richness and evenness into account. In this study, we computed for each community the exponential of the Shannon index based on the total community species richness and evenness.	$\mathrm{H}' = -\sum_i (p_i * \ln(p_i))$
Species richness	Total number of species seen at least one year during the time series. Species richness is computed for each site and each dataset.	
Weighted or non-weighted Mean Pairwise Distance (MPD)	Reflects the phylogenetic diversity inside a community. The standard MPD is computed as the mean pairwise phylogenetic distance between species in a community. The weighted MPD is computed the same way, but distances are weighted by the product of the abundances of the species that constitute the pair	

Footnote: With CV the coefficient of variation of community abundances, μ the community mean abundance, σ the standard deviation of community abundance, CV_w the weighted mean coefficient of variation of population abundance in a community, μ_i the mean abundance of the population *i* in a community and σ_i its standard deviation, φ the synchrony between population abundance fluctuation in a community, H' the Shannon index, *n* the species of a community and p_i the proportion of the abundance of the species *i* in its community.

Supplementary Table 3. Standardized coefficients from the Structural Equation Models on the stability of bat, bird and butterfly communities for the three buffer sizes. Est. stands for estimates. SE for standard errors. P-val. for *p*-value.

		Bats								
		250m buffer radius<br>Fischer C= 9.31<br>*p*-value= 0.503<br>AIC= 65.31			500m buffer radius<br>Fischer C= 9.02<br>*p*-value= 0.531<br>AIC= 65.02			1000m buffer radius<br>Fischer C= 10.51<br>*p*-value= 0.397<br>AIC=66.51		
Response	Predictor	Est.	SE	P-val.	Est.	SE	P-val.	Est.	SE	P-val.
Species diversity	Agricultural intensity gradient	0.119	0.091	0.192	0.097	0.091	0.290	0.141	0.094	0.135
	Urban gradient	0.298	0.085	0.001	0.283	0.085	0.001	0.309	0.088	0.001
Phylogenetic diversity	Agricultural intensity gradient	-0.187	0.088	0.035	-0.192	0.087	0.030	-0.211	0.090	0.021
	Urban gradient	-0.181	0.080	0.026	-0.188	0.080	0.020	-0.174	0.084	0.040
Population stability	Agricultural intensity gradient	-0.246	0.091	0.008	-0.254	0.091	0.006	-0.237	0.096	0.015
	Urban gradient	-0.102	0.091	0.261	-0.105	0.090	0.244	-0.080	0.093	0.390
	Phylogenetic diversity	0.061	0.082	0.459	0.059	0.082	0.476	0.065	0.082	0.429
	Species richness	-0.190	0.081	0.021	-0.192	0.081	0.019	-0.194	0.081	0.018
Population asynchrony	Agricultural intensity gradient	0.031	0.086	0.721	0.088	0.086	0.308	0.140	0.090	0.121
	Urban gradient	-0.111	0.085	0.197	-0.039	0.085	0.648	0.078	0.087	0.375
	Phylogenetic diversity	0.222	0.077	0.005	0.243	0.077	0.002	0.260	0.077	0.001
	Species richness	0.412	0.077	0.192	0.391	0.076	<0.001	0.362	0.076	<0.001
Community stability	Population asynchrony	0.633			0.633			0.633		
	Population stability	0.687			0.687			0.687		

		Birds								
		1000m buffer radius<br>Fischer C= 5.96<br>*p*-value= 0.819<br>AIC= 65.96			1250m buffer radius<br>Fischer C= 5.56<br>*p*-value= 0.851<br>AIC= 65.56			1500m buffer radius<br>Fischer C= 5.15<br>*p*-value= 0.881<br>AIC= 65.15		
Response	Predictor	Est.	SE	P-val.	Est.	SE	P-val.	Est.	SE	P-val.
Species diversity	Agricultural intensity gradient	-0.229	0.061	<0.001	-0.210	0.062	0.001	-0.187	0.063	0.003
	Urban gradient	-0.117	0.056	0.038	-0.138	0.057	0.017	-0.163	0.058	0.006
Phylogenetic diversity	Agricultural intensity gradient	0.115	0.066	0.083	0.150	0.066	0.023	0.178	0.066	0.007
	Urban gradient	-0.019	0.063	0.759	-0.017	0.064	0.796	-0.016	0.064	0.799
Population stability	Agricultural intensity gradient	-0.414	0.063	<0.001	-0.409	0.064	<0.001	-0.397	0.065	<0.001
	Urban gradient	0.071	0.057	0.218	0.079	0.059	0.176	0.081	0.060	0.179
	Phylogenetic diversity	0.023	0.055	0.680	0.034	0.055	0.540	0.041	0.056	0.462
	Species richness	-0.040	0.061	0.512	-0.030	0.061	0.623	-0.017	0.062	0.786
Population asynchrony	Agricultural intensity gradient	0.014	0.061	0.816	0.011	0.061	0.857	0.017	0.061	0.784
	Urban gradient	0.025	0.059	0.673	0.041	0.059	0.494	0.050	0.059	0.399
	Phylogenetic diversity	0.033	0.059	0.573	0.033	0.059	0.581	0.030	0.059	0.607
	Species richness	0.360	0.060	<0.001	0.363	0.060	<0.001	0.366	0.060	<0.001
Community stability	Population asynchrony	0.722			0.722			0.722		
	Population stability	0.500			0.500			0.500		

		Butterflies								
		250m buffer radius<br>Fischer C= 20.49<br>*p*-value= 0.025<br>AIC= 72.49			500m buffer radius<br>Fischer C= 19.44<br>*p*-value= 0.035<br>AIC= 71.44			1000m buffer radius<br>Fischer C= 21.58<br>*p*-value= 0.017<br>AIC= 73.58		
Response	Predictor	Est.	SE	P-val.	Est.	SE	P-val.	Est.	SE	P-val.
Species diversity	Agricultural intensity gradient	-0.085	0.084	0.316	-0.131	0.082	0.111	-0.184	0.079	0.021
	Urban gradient	-0.297	0.084	0.001	-0.366	0.082	<0.001	-0.439	0.079	<0.001
Phylogenetic diversity	Agricultural intensity gradient	-0.049	0.062	0.429	-0.059	0.064	0.358	-0.045	0.067	0.510
	Urban gradient	0.227	0.062	<0.001	0.258	0.061	<0.001	0.239	0.062	<0.001
Population stability	Agricultural intensity gradient	0.085	0.081	0.294	0.047	0.083	0.575	0.006	0.084	0.948
	Urban gradient	-0.374	0.085	<0.001	-0.366	0.089	<0.001	-0.394	0.090	<0.001
	Phylogenetic diversity	0.192	0.082	0.020	0.202	0.084	0.019	0.191	0.084	0.025
	Species richness	0.158	0.084	0.062	0.133	0.087	0.131	0.095	0.090	0.294
Population asynchrony	Agricultural intensity gradient	0.139	0.085	0.106	0.176	0.086	0.042	0.186	0.087	0.035
	Urban gradient	0.045	0.089	0.616	0.102	0.091	0.268	0.118	0.094	0.212
	Phylogenetic diversity	0.254	0.086	0.004	0.257	0.087	0.004	0.268	0.087	0.003
	Species richness	0.247	0.088	0.006	0.283	0.090	0.002	0.299	0.093	0.002
Community stability	Population asynchrony	0.637			0.637			0.637		
	Population stability	0.710			0.710			0.710		

Supplementary Table 4. GeneBank accession numbers of genes used to generate the phylogeny of butterflies.

Species	Gene				
	COI	wingless	EF-1α	GAPDH	RpS5
Aglais urticae	HQ003951	AF412777	AY248811	FJ639522	FJ639577
Aporia crataegi	HM393180	EU141242	AY870529	EU141496	EU141394
Argynnis paphia	FJ663298	AY090133	GU372616	EU141519	EU141421
Brintesia circe	HQ004137	DQ338729	DQ339020	EU141474	EU141370
Cacyreus marshalli	AY556966	GQ128914	GQ128703		
Coenonympha pamphilus	GU707174	DQ338637	DQ338920	EU528385	EU528428
Inachis io	FJ663678	AF412766	AY248810	FJ639521	FJ639576
Iphiclides podalirius	GU559736	DQ351129	AF173413		
Macroglossum stellatarum	JF415393	EU479508	EU479296		
Maniola jurtina	FJ663756	AY090147	AY090180	EU141481	EU141376
Papilio machaon	HQ004884	AY569124	AF044828		
Pararge aegeria	GU655008	DQ176340	DQ338913	EU141476	EU141372
Vanessa atalanta	FJ664079	HQ734826	AY090187	HQ734959	HQ735034
Vanessa cardui	HQ990383	HQ734828	HQ734947	HQ734971	HQ735047

Supplementary Table 5. Variables used to describe the landscape surrounding the study sites. The urban, cropland, heterogenous agricultural, woodland and semi-natural open areas. CLC is for Corine Land Cover.

Data	Description	CLC Code	Units
Soil sealing	Sealed soil		m^2
Agricultural inputs	Fertilizers		k€
	Pesticides		k€
	Livestock food		k€
	Veterinarian medics		k€
Landscape complexity	Shannon index based on CLC land-uses	111-524	
Urban area	Artificial surfaces	111-142	m^2
Cropland area	Arable land	211-213	m^2
	Permanent crops	221-223	m^2
Heterogeneous agricultural areas	Pastures	231	m^2
	Heterogeneous agricultural areas	241-244	m^2
Woodland area	Forests	311-313	m^2
Semi-natural open area	Scrub and/or herbaceous vegetation associations	321-324	m^2

References

1. Shi, J. J. & Rabosky, D. L. Speciation dynamics during the global radiation of extant bats. *Evolution* **69**, 1528–1545 (2015).
