# Supplementary material for: Urbanization and agricultural intensification destabilize animal communities differently than diversity loss
Source: Nat Commun. 2020 Jun 1;11:2686. doi: 10.1038/s41467-020-16240-6 (PMC7264125; doi:10.1038/s41467-020-16240-6)
Supplement: Supplementary file 3 — Reporting Summary [file 41467_2020_16240_MOESM3_ESM.pdf]

nature research

Corresponding author(s): Théophile Olivier

Last updated by author(s): 31/03/2020

# Reporting Summary

Nature Research wishes to improve the reproducibility of the work that we publish. This form provides structure for consistency and transparency in reporting. For further information on Nature Research policies, see Authors & Referees and the Editorial Policy Checklist .

## Statistics

For all statistical analyses, confirm that the following items are present in the figure legend, table legend, main text, or Methods section.

| n/a | Confirmed | |
|---|---|---|
| ☐ | ☒ | The exact sample size ($n$) for each experimental group/condition, given as a discrete number and unit of measurement |
| ☐ | ☒ | A statement on whether measurements were taken from distinct samples or whether the same sample was measured repeatedly |
| ☐ | ☒ | The statistical test(s) used AND whether they are one- or two-sided<br>*Only common tests should be described solely by name; describe more complex techniques in the Methods section.* |
| ☐ | ☒ | A description of all covariates tested |
| ☐ | ☒ | A description of any assumptions or corrections, such as tests of normality and adjustment for multiple comparisons |
| ☐ | ☒ | A full description of the statistical parameters including central tendency (e.g. means) or other basic estimates (e.g. regression coefficient) AND variation (e.g. standard deviation) or associated estimates of uncertainty (e.g. confidence intervals) |
| ☒ | ☐ | For null hypothesis testing, the test statistic (e.g. $F$, $t$, $r$) with confidence intervals, effect sizes, degrees of freedom and $P$ value noted<br>*Give P values as exact values whenever suitable.* |
| ☒ | ☐ | For Bayesian analysis, information on the choice of priors and Markov chain Monte Carlo settings |
| ☒ | ☐ | For hierarchical and complex designs, identification of the appropriate level for tests and full reporting of outcomes |
| ☒ | ☐ | Estimates of effect sizes (e.g. Cohen's $d$, Pearson's $r$), indicating how they were calculated |

*Our web collection on statistics for biologists contains articles on many of the points above.*

## Software and code

Policy information about availability of computer code

| | |
|---|---|
| Data collection | We used R software version 3.5.1, Syrinx 2.6, Tree Annotator 1.7.5, CodonCode Aligner 6.0.2, BEAST 1.8.1 |
| Data analysis | We used R software version 3.5.1 with R packages vegan (2.5-1), nlme (3.1-137), picante (1.8), ape (5.1), ade4 (1.7-11), lmerTEST (3.0-1), PiecewiseSEM (1.2.0) |

For manuscripts utilizing custom algorithms or software that are central to the research but not yet described in published literature, software must be made available to editors/reviewers. We strongly encourage code deposition in a community repository (e.g. GitHub). See the Nature Research guidelines for submitting code & software for further information.

## Data

Policy information about availability of data

All manuscripts must include a data availability statement. This statement should provide the following information, where applicable:

- Accession codes, unique identifiers, or web links for publicly available datasets
- A list of figures that have associated raw data
- A description of any restrictions on data availability

The datasets and codes that support the findings of this study are available in Zenodo with the identifier [https://doi.org/10.5281/zenodo.3736101]. Community raw data come from citizen science programs hosted by the Vigie Nature program [http://vigienature.mnhn.fr/]. For bats we used the French bat-monitoring program [http://www.vigienature.fr/fr/chauves-souris]. For birds we used the French Breeding Bird Survey [http://www.vigie-plume.fr/]. For butterflies we used the French garden butterfly observatory [http://www.vigienature.fr/fr/operation-papillons].

Data used to compute land-use areas and landscape complexity are available on the Corine Land Cover website [https://www.data.gouv.fr/fr/datasets/corine-land-cover-occupation-des-sols-en-france/]. Data used to compute sealed soil are available on the EEA website [http://www.eea.europa.eu/data-and-maps/explore-interactive-maps/european-soil-sealing-v2]. Data used to compute the agricultural inputs are available on the AGRESTE website [http://agreste.agriculture.gouv.fr/enquetes/reseau-d-information-comptable/].

# Field-specific reporting

Please select the one below that is the best fit for your research. If you are not sure, read the appropriate sections before making your selection.

- [ ] Life sciences
- [ ] Behavioural & social sciences
- [x] Ecological, evolutionary & environmental sciences

For a reference copy of the document with all sections, see nature.com/documents/nr-reporting-summary-flat.pdf

# Ecological, evolutionary & environmental sciences study design

All studies must disclose on these points even when the disclosure is negative.

| | |
|---|---|
| Study description | In this study we analyzed the inter-annual abundance fluctuations of 152 bat, 269 bird and 130 butterfly communities across France, monitored following standardized protocols over six, 17 and 11 years, respectively, to quantify how community stability is affected by local diversity and habitat degradation. |
| Research sample | Data come from three citizen science programs, "Vigie Chiro" (bat data), "Opération Papillons" (butterfly data) and "Suivi Temporel des Oiseaux Communs" (bird data), developed at Vigie Nature at the National Museum of Natural History of Paris. These three taxa represent three different animal groups (insects, birds and mammals) with differences in life history such as life expectancy, ecological traits such as food resources used and sensitiviy to environmental conditions and perturbations. |
| Sampling strategy | Regarding community species diversity, we selected sites with at least two species observed during the survey periods, to take account of only communities and not isolated populations for a given taxa. For bats, we used the 7 species recorded. For birds we used data about 75 common species for which the amount of data available allows an accurate estimation of population dynamic. For butterflies, participants identify and count Lepidoptera in their own garden, from a closed list of 28 common species or species groups (27 butterflies and one common diurnal moth, Macroglossum stellatarum). Since some of the taxa targeted by this scheme group several look-alike species (species groups), we only kept the 13 butterflies and 1 moth identified at species level for our analyses. |
| Data collection | Data collection have been performed by volunteers of the Vigie Nature programs.<br>For bats, volunteers record bat activity using ultrasound recorder while driving at a constant low-speed (25 ± 5 km/h) along 30 km circuits. Bat activity is recorded through echolocation calls with ultrasound detectors connected to a digital recorder. Volunteers are trained to classify echolocation calls to the most accurate taxonomic level using Syrinx 2.6. Data validation is done by program coordinators at the Muséum national d'Histoire naturelle for recordings with uncertain identification. The abundance of each bat species in a 2-km road transect is defined as the number of bat pass per species (a bat pass corresponds to a trigger of the bat detector in time expansion).<br>For birds, keen birdwatchers count birds annually in a given plot. Plots are squares of 2x2km2 randomly selected by the national coordinator, within which the surveyor places 10 points separated by at least 300m, in order to cover all the habitats present in the plot. Each plot is surveyed twice a year, the first session between April 1st and May 8th, the second between May 9th and June 30th, with at least four weeks between both sessions. Surveying dates must be the same (+/- 5 days) every year, and counting takes place in the morning, starting 30 min after sunrise, with points always visited in the same order. At each point, the volunteer spends 5 minutes recording all birds seen or heard.<br>For butterflies, participants identify and count Lepidoptera in their own garden, from a closed list of common species or species groups. For each species, abundances are recorded monthly, as the maximum number of butterflies seen simultaneously during the month. |
| Timing and spatial scale | Regarding time series length, we used for each sites all available years, in the whole France. This selection corresponds to data between 2006 and 2012 for bats, 2001 and 2017 for birds and between 2007 and 2018 for butterflies. This leads to 65, 7 and 80 sites with time series of respectively 4, 5, and 6 years for bats; 3, 14, 37, 32, 29, 33, 49, 30, 30 and 12 sites with time series of respectively 8, 9, 10, 11, 12, 13, 14, 15, 16 and 17 years for birds; and 34, 12, 10, 17 and 57 sites with time series of respectively 7, 8, 9, 10 and 11 years for butterflies. We choose the maximum number of years for each site to increase the quality of ou population and community variability measure. However, this choice gave us time series with gaps, not always for the same years across sites.<br><br>To test the robustness of our results to the presence of gaps in the time series, we also ran our analyses on two subsets of the dataset that included only time series of the same duration and with no missing year. The first subset was restricted to the longest fully overlapping observation period with no gap common to all sites, leading to times series of 4, 8 and 7 years for bats, butterflies and birds, respectively. The second one was restricted to sites having the longest fully overlapping observation period with no gap, leading to time series of 5, 12 and 11 years, for bats, birds and butterflies, respectively.<br><br>Finally we did not restricted the spatial area of where the used sites were located, to integrate a wider range of environmental conditions in our analyses. |
| Data exclusions | As mentioned in the sampling strategy section, we only used sites with at least two species, to only work on communities. This critieria was pre-established. |
| Reproducibility | To test the robustness of our results, we used three species diversity metrics, a Shannon index, the species richness and the Chao index. The use of either Shannon index, species richness or Chao index did not change qualitatively the results of the analysis. To test the robustness of our results to the presence of gaps in the time series, we ran our analyses on three subsets of the dataset. The analysis of the three datasets gave qualitatively similar results, confirming the robustness of our results to both the presence of gaps and the number of communities studied. See details in Methods and Supplementary material sections. |

| | |
|---|---|
| Randomization | Because we performed only linear regressions, randomization was not relevant to our study. See details in Methods section. |
| Blinding | Blinding was not relevant to our study. See details in Methods section. |

Did the study involve field work? ☐ Yes ☒ No

# Reporting for specific materials, systems and methods

We require information from authors about some types of materials, experimental systems and methods used in many studies. Here, indicate whether each material, system or method listed is relevant to your study. If you are not sure if a list item applies to your research, read the appropriate section before selecting a response.

## Materials & experimental systems

| n/a | Involved in the study |
|---|---|
| ☒ | ☐ Antibodies |
| ☒ | ☐ Eukaryotic cell lines |
| ☒ | ☐ Palaeontology |
| ☒ | ☐ Animals and other organisms |
| ☒ | ☐ Human research participants |
| ☒ | ☐ Clinical data |

## Methods

| n/a | Involved in the study |
|---|---|
| ☒ | ☐ ChIP-seq |
| ☒ | ☐ Flow cytometry |
| ☒ | ☐ MRI-based neuroimaging |
